# B-waves in noninvasive capacitance signal correlate with B-waves in ICP

**DOI:** 10.1007/s00701-025-06604-6

**Published:** 2025-07-08

**Authors:** Andreas Spiegelberg, Andrea Boraschi, Ramy Amirah, Katharina Wolf, Mukesch Shah, Laura Krismer, Jürgen Beck, Vartan Kurtcuoglu

**Affiliations:** 1 The Interface Group, Department of Physiology, University of Zurich, Zurich, Switzerland; 2 Department of Neurosurgery, Medical Center, University of Freiburg, Freiburg, Germany; 3 Zurich Center for Integrative Human Physiology, University of Zurich, Zurich, Switzerland; 4 Neuroscience Center Zurich, University of Zurich, Zurich, Switzerland

To the Editor

We thank Drs. Batchu and Thomas for their thoughtful commentary [1] on our recent technical report [5] that explores the correlation between B-waves in intracranial pressure (ICP) and oscillations of the same frequency in W, a noninvasively measured capacitance signal of the head.

As they correctly note, and as we also state in our report, the study was limited to induced B-waves under controlled conditions and did not include monitoring during spontaneous physiological states such as REM sleep. While not included in the publication, we did collect data in the same patient cohort under different body positions; however, these modulations did not consistently evoke B-waves in ICP. In a recent pilot study on healthy volunteers, we observed that W reflects instantaneous intracranial volume composition changes in response to head-down tilting and jugular vein compression [2]. Monitoring during sleep, where spontaneous B-waves are more likely to emerge, was outside the scope of the study. Accordingly, we refrained from proposing immediate clinical applications, instead suggesting that the W signal's potential as a noninvasive triage tool should be evaluated further.

The correspondents further correctly note that the craniospinal pressure–volume relation is nonlinear. Therefore, linear regression is not strictly valid for comparing B-wave amplitudes in ICP and W, given that the former reflects pressure and the latter volume-related fluctuations. While an exponential model may appear more intuitive – consistent with Marmarou’s concept [4] – it is also not strictly appropriate in our context, as each patient has an individual pressure–volume curve characterized, at minimum, by their proper equilibrium pressure and compliance coefficient. An exponential model would have been the correct choice for assessing B-wave amplitudes in individual patients across different compliance states, for example provoked through a staged infusion protocol allowing prolonged observation at varying pressure levels. Such an approach was not feasible within the ethical and practical constraints of our study. Therefore, having had the choice between two regression models, neither of which was strictly valid, we opted to choose the simpler model in line with the principle of parsimony in model selection [3].

We appreciate Drs. Batchu and Thomas’ constructive suggestions and agree that further research is needed to define the potential clinical utility of W in noninvasive intracranial monitoring.

## Data Availability

No datasets were generated or analysed during the current study.
